# The microRNA Let-7f Induces Senescence and Exacerbates Oxidative Stress in Retinal Pigment Epithelial Cells

**DOI:** 10.3390/antiox13060646

**Published:** 2024-05-25

**Authors:** Christina Ortiz, Houda Tahiri, Chun Yang, Claudia Gilbert, Carl Fortin, Pierre Hardy

**Affiliations:** 1 Departments of Pharmacology and Physiology, Faculty of Medicine, Université de Montréal, Montréal, QC H3T 1J4, Canada; christina.ortiz@cnrc-nrc.gc.ca; 2 CHU Sainte-Justine Research Center, Université de Montréal, Montréal, QC H3T 1C5, Canada; houda.tahiri.hsj@ssss.gouv.qc.ca (H.T.); cyang_09@yahoo.com (C.Y.); claudia.gilbert2.hsj@ssss.gouv.qc.ca (C.G.); carl.fortin.hsj@ssss.gouv.qc.ca (C.F.); 3 Department of Pediatrics, Faculty of Medicine, Université de Montréal, Montréal, QC H3T 1J4, Canada

**Keywords:** dry age-related macular degeneration, microRNA let-7f, oxidative stress, senescence

## Abstract

This study aims to investigate the role of microRNA let-7f in the dysfunction and degeneration of retinal pigment epithelium (RPE) cells through the induction of senescence and oxidative stress. Furthermore, we explore whether let-7f inhibition can protect these cells against sodium iodate (SI)-induced oxidative stress. Oxidative stress and let-7f expression are reciprocally regulated in retinal pigment epithelial cells. Overexpression of let-7f in ARPE-19 cells induced oxidative stress as demonstrated by increased reactive oxygen species (ROS) production as well as senescence. Inhibition of let-7f successfully protected RPE cells from the detrimental effects induced by SI. In addition, let-7f overexpression induced RPE cellular dysfunction by diminishing their migratory capabilities and reducing the phagocytosis of porcine photoreceptor outer segments (POS). Results were further confirmed in vivo by intravitreal injections of SI and let-7f antagomir in C57BL/6 mice. Our results provide strong evidence that let-7f is implicated in the dysfunction of RPE cells through the induction of senescence and oxidative injury. These findings may help to uncover novel and relevant processes in the pathogenesis of dry AMD.

## 1. Introduction

The retinal pigment epithelium (RPE) is a specialized monolayer of cells located at the outermost layer of the retina [1,2]. RPE cells are multifunctional and essential for maintaining proper vision. In addition to forming the blood–retinal barrier, playing a role in the visual cycle and absorbing excess scattered light, RPE cells are the caretakers of photoreceptor cells. RPE cells are responsible for the phagocytosis and clearance of shed photoreceptor tips and are thus fundamental in maintaining photoreceptor survival and functioning [3,4]. RPE cells must meet high energy demands to perform their many functions, rendering them particularly susceptible to oxidative injury. The excessive production of reactive oxygen species (ROS) in the RPE is usually counterbalanced by an efficient endogenous antioxidant defense mechanism. However, with aging, the strength of this defense system declines, leading to progressive dysfunction and the eventual degeneration or death of RPE cells. When coupled with additional oxidant-promoting stressors, such as smoking, obesity and genetics, age-associated damage to the RPE may lead to the development of various ocular diseases such as age-related macular degeneration (AMD) [5,6].

In developed countries, AMD is the primary cause of blindness and visual impairment amongst older people. There exist two forms of this disease: dry AMD and wet AMD. Wet AMD, also known as neovascular AMD, can progress rapidly causing vision loss within days or weeks due to the growth of abnormal blood vessels underneath the retina which damage the RPE monolayer. Although it is the less severe form of the disorder, approximately 90% of all people with AMD suffer from the dry form, which is characterized by the accumulation of sub-RPE lipid waste deposits, also known as drusen, which induce atrophy of the RPE monolayer. To date, there exist no approved treatments capable of going beyond merely slowing the progression of this disease [7,8]. Decades of research have indicated that chronic oxidative stress at the RPE is a major contributing factor to the pathogenesis of dry AMD [6,7,9]. Another key player in the development and progression of dry AMD is senescence. Senescent RPE cells were first identified in older primates by Mishima et al. in 1999 [10]. More recent studies have revealed that oxidative stress induces premature senescence in RPE cells, thereby further exacerbating cellular dysfunction [11,12,13].

MicroRNAs are small non-coding RNAs that can silence gene expression by interacting with the 3′-UTR of target mRNAs, making them master regulators of post-transcriptional gene expression in various cellular pathways [14]. The first discovered miRNAs, the let-7 family, are comprised of 12 members (let-7a-1, 7a-2, 7a-3, 7b, 7c, 7d, 7e, 7f-1, 7f-2, 7g, 7i and mir-98) and are highly conserved across species. In humans, this family of miRNAs has well-established functions in cellular differentiation, cell cycle regulation and tumor suppression [15,16]. Additionally, they have been associated with the regulation of aging, oxidative stress and senescence in various cell types, including many related to ocular tissues [17,18,19,20,21,22]. A recent study by Wooff et al. has identified four members of the let-7 family, including let-7f, amongst the top ten most abundant miRNAs in extracellular vesicles released by photo-oxidative damaged retinas [23]. Despite substantial evidence linking let-7 miRNAs to age-related retinal disorders, their functional roles in the RPE remain essentially unexplored. In the present study, we examined the in vitro and in vivo expression of let-7f in RPE cells under sodium iodate (SI)-induced oxidative stress. Our findings provide novel evidence for the implication of let-7f in the dysfunction and the induction of senescence in RPE cells.

## 2. Materials and Methods

### 2.1. Animals

The animal protocol (#2021-3125) was approved by the CHU Sainte-Justine Research Center Animal Welfare Committee CIBPAR and followed the guidelines of the Canadian Council on Animal Care and the Guide for the Care and Use of Laboratory Animals published by the US National Institutes of Health. Six eight-week-old female C57BL/6J mice were purchased from Charles River (St. Constant, QC, Canada) and housed in a pathogen-free environment with a 12:12 h light/dark cycle with free access to chow and water.

### 2.2. In Vivo Retinal Degeneration Mouse Model

The mouse model of sodium iodate (SI)-induced retinal degeneration is a well-established, widely used model for pro-oxidant-induced RPE degeneration [24,25]. Before injection, SI was dissolved in saline and sterilized. Mice received a single systemic tail-vein injection of 50 mg/kg. One group of mice was injected intravitreously with 1 µg let-7f antagomir (hsa-let-7f-5p miRNA antagomir, Applied Biological Materials, Richmond, BC, Canada) using Invivofectamine^®^ 3.0 (Thermo Scientific, Rockford, IL, USA), 24 h prior to SI injection. As an internal control, the contralateral eye was injected with 1 µg scrambled miRNA. The mice were sacrificed and their eyes were collected on days 0, 3, 5, 7 and 10. A minimum of three mice was used for each treatment group.

### 2.3. Retinal Histology

After sacrifice, mice were enucleated and whole eyes were fixed in 4% PFA for 1 h at room temperature, washed in PBS and cryoprotected overnight at 4 °C in a 30% sucrose solution. Eyes were then embedded and snap frozen in O.C.T (Sakura Finetek, Torrance, California, USA). Sagittal 10 µm retinal cryosections were prepared with a Cryostat Epredia (CryoStar NX50, Thermo Scientific, Rockford, IL, USA) microtome. Sections were stained with hematoxylin and eosin (H&E) following standard protocols. Images were obtained using a brightfield Leica DMi8 inverted microscope (Leica Microsystems, Mannheim, Germany) at 20×.

### 2.4. RPE/Eyecup Wholemount Preparation and Labeling

After sacrifice, mice were enucleated and whole eyes were fixed in 4% PFA for 1 h at room temperature. Under a dissecting microscope, eyes were cleaned of extraocular tissue and carefully dissected to remove the anterior segment (i.e., cornea, iris, ciliary body and lens). Four radial cuts were made toward the optic nerve to facilitate flattening of the posterior eyecup, resulting in a petal-shaped structure, and then the neural retina was gently peeled away, exposing the intact RPE monolayer. RPE/eyecups were then carefully deposited into 96-well plates, permeabilized with 0.1% Triton X-100 at room temperature for 1 h and incubated at 4 °C overnight with Phalloidin-iFluor-488 (1:100, Abcam) in 1% bovine serum albumin (BSA). RPE/eyecups were transferred onto slides with the RPE layer facing up using a plastic transfer pipette. Excess liquid on the slide, around the tissue was absorbed using a kimwipe and the ends of the wholemount were gently extended to prevent folding of the petals prior to addition of mounting liquid (ProLong Diamond Antifade, Thermo Scientific, Rockford, IL, USA) and coverslip. Wholemount images were generated using a laser scanning confocal microscope (Leica TCS SP8-STED, Leica Microsystems, Mannheim, Germany) and Z-stacking. For each wholemount, four images (one image per petal, ~300 µm × 300 µm) were used for analysis. Damaged area was measured using Fiji/ImageJ software v1.54h.

### 2.5. Cell Culture

Human RPE cells (ARPE-19) were purchased from American Type Culture Collection (ATCC, CRL-2302) and cultured in Dulbecco’s modified Eagle’s medium (DMEM)/F12 (Gibco, Thermo Scientific, Rockford, IL, USA) supplemented with 10% fetal bovine serum, 100 U/mL penicillin and 100 µg/mL streptomycin and maintained at 37 °C in a humidified 5% CO_2_ atmosphere. Only cell passages 6 through 13 were used for the following experiments unless otherwise indicated.

### 2.6. Cell Transfection and Treatments

With a TransIT-X2^®^ Dynamic Delivery System (Mirus Bio LLC, Madison, WI, USA), ARPE-19 cells (80% confluency) were transfected with 50 nM hsa-let-7f-5p mirVana miRNA mimic (Ambion, Thermo Scientific, Rockford, IL, USA) for 48 h to induce the overpression of let-7f. To inhibit let-7f expression, cells were transfected with 20 nM hsa-let-7f-5p miRNA antagomir (Applied Biological Materials, Solana Beach, CA, USA) for 48 h. For each experiment, control cells were transfected with their respective scrambled miRNA (Sc). Another group of cells was treated with 5 mM SI (Sigma-Aldrich, Oakville, ON, Canada) 1 h after transfection with let-7f antagomir. A 50 mM working solution of SI was freshly prepared by dissolving in DMEM/F12 media and sterilized through a 0.22 µm syringe filter. SI added to the cells every 24 h to ensure stability.

### 2.7. Cell Viability Assays

ARPE-19 cells were plated ($1\times 10^{4}$ cells/well) onto 96-well black plates and treated according to the procedure described above. Cell viability was quantified using PrestoBlue^®^ assay (Invitrogen, Waltham, MA, USA, Thermo Fisher Scientific, Fresno, CA, USA) by adding 10 µL/well of the reagent and incubating in the dark at 37 °C for 30 min. Fluorescence was read using a microplate reader (CLARIOstar, BMG Labtech; excitation 535 nm, emission 615 nm).

### 2.8. [^3^H]-Thymidine Incorporation Proliferation Assays

ARPE-19 cells were plated ($5\times 10^{4}$ cells/well) onto 24-well plates and the incorporation of radioactive thymidine into DNA was used to quantify the rate of DNA synthesis. Briefly, 10 µCi [^3^H]-thymidine was added per well, and cells were treated as described above. Thymidine incorporation was measured 48 h after cell transfections and treatments using a liquid scintillation counter (PerkinElmer, Waltham, MA, USA).

### 2.9. Scratch Wound Migration Assays

Cell migration was evaluated by plating ARPE-19 cells ($5\times 10^{4}$ cells/well) onto 24-well plates and immediately prior to transfection, the narrow end of a p1000 tip was used to scratch a straight line diagonally through the cells across the center of the whole well. Cells were washed with PBS and fresh media was added to remove cellular debris. Images of the center of the well, where scratch was more linear, were taken at time points 0 (immediately after the scratch wound), 24, 48 and 72 h at 4X using live-cell imaging IncuCyte™ system (Essen Bioscience, Ann Arbor, MI, USA). Scratch wound area, represented by the total area of blue-outlined sections, was calculated using Fiji/ImageJ software v1.54h. The percent of healed gap wound area was calculated by the reduction in wound area at 72 h compared to wound area at time 0.

### 2.10. Isolation and Fluorescence Labeling of Photoreceptor Outer Segments

Photoreceptor outer segments (POS) were isolated and purified from 20 fresh adult porcine eyes purchased from Abattoir Campbell (Saint-Sébastien, QC, Canada), according to a modified method [26]. Briefly, eyes were dissected, and retinas were homogenized in a solution containing 20% sucrose, 20 mM Tris/Acetate pH 7.2, 2 mM MgCl_2_, 10 mM glucose and 5 mM taurine. Homogenized solution was filtered 3 times through gauze to remove large debris. Crude isolate was carefully overlaid over a linear gradient of 25–60% sucrose and ultra-centrifuged at 25,000 rpm (CP90NX, Hitachi, Tokyo, Japan) for 48 min at 4 °C. POS fraction was identified as a sharp single pink band in the upper part of the gradient. POS were collected, washed and resuspended in 2.5% sucrose in DMEM. POS were counted with a hemocytometer. POS resuspension was diluted to $1\times 10^{8}$ POS/mL in 2.5% sucrose in DMEM, aliquoted and stored at −80 °C. A portion of collected POS was labeled with fluorescein-5-isothiocyanate (FITC) by incubating in a 2 mg/mL FITC solution for 1 h at room temperature in the dark with rotation to ensure labeling of all POS. Labeled-POS were washed twice by extensive resuspension in a solution of 10% sucrose, 20 mM phosphate buffer pH 7.2 and 5 mM taurine to remove unbound FITC. Labeled-POS were counted, resuspended and aliquoted as described for unlabelled-POS.

### 2.11. Phagocytosis Assays

For phagocytic activity assays, ARPE-19 cells were plated ($1\times 10^{4}$ cells/well) onto 96-well black plates in complete medium until 100% confluence was achieved, followed by two weeks of sub-culturing in complete media reduced to 1% FBS to achieve mature cell polarization and tight-junction formation, indicated by cells forming a highly dense hexagonal shaped monolayer and displaying increased pigmentation. After transfection, mature and polarized cells were fed with FITC-POS (10 POS/cell) and incubated for 6 h at 37 °C. Unbound POS were removed by extensive washing with PBS. The fluorescence of phagocytosed FITC-POS was recorded with a fluorescent microplate reader (CLARIOstar, BMG Labtech; excitation 483 nm, emission 530 nm).

### 2.12. Immunofluorescence Staining

ARPE-19 cells ($5\times 10^{4}$ cells/well) were grown on coverslips inserted into 24-well plates. After treatment, cells were fixed in 4% PFA for 15 min followed by permeabilization and blocking in 0.1% Triton X-100, 2% normal goat serum and 1% BSA in PBS for 1 h at room temperature. Cells were then incubated overnight at 4 °C with rabbit anti-human Ki67 (1:500, Abcam, Cambridge, UK) in 1% BSA, washed three times in PBS, incubated with secondary antibody AlexaFluor-594 (1:500, Molecular Probes, Eugene, OR, USA) for 1 h at room temperature and then mounted onto slides with mounting liquid containing DAPI (ProLong Diamond Antifade, Thermo Scientific, Rockford, IL, USA). Images of 3 random frames were captured at 10× using a Leica DMi8 (Leica Microsystems, Mannheim, Germany) inverted wide-field fluorescence microscope and analyzed with Fiji/ImageJ software v1.54h. The percent of Ki67 positive cells was calculated based on the number of Ki67 positive cells divided by the number of DAPI positive cells.

### 2.13. Senescence-Associated β-Galactosidase Assays

ARPE-19 cells were plated ($5\times 10^{4}$ cells/well) onto 24-well plates, transfected and stained with Senescence-Associated β-Galactosidase (SA-β-gal) Staining Kit (Cell Signaling, #9860) in which the activity of lysosomal β-galactosidase enzyme at pH 6, cleaves and converts substrate X-gal into a visually detectable oxidized blue product [27]. Briefly, cells were fixed in provided 1X fixative solution for 15 min at room temperature. After washing three times with PBS, cells were incubated in freshly prepared β-gal staining solution (1 mg/mL X-gal, pH 6) overnight at 37 °C in a dry incubator (no CO_2_). Ensuring a final pH of 6.0 for the β-gal staining solution was crucial in preventing false positives or false negatives. Cells were washed, rinsed in methanol and air-dried before microscopic examination. Brightfield images of at least 100 cells per frame (10×), were taken with a Leica DMi8-inverted microscope (Leica Microsystems, Mannheim, Germany). Senescent cells were identified by the development of blue coloration as well as an enlarged, flattened cell morphology.

### 2.14. Measurement of Intracellular Reactive Oxygen Species Production

Intracellular production of ROS was measured with a 5-(and-6)-chloromethyl-2′,7′-dichlorodihydrofluorescein diacetate fluorescent probe (CM-H_2_DCFDA, Thermo Scientific, Rockford, IL, USA). ARPE-19 cells were plated ($1\times 10^{4}$ cells/well) onto 96-well black plates, transfected as described above and incubated with 5 µM CM-H_2_DCFDA for 30 min at 37 °C. Fluorescence intensity was measured with a microplate reader (CLARIOstar, BMG Labtech; excitation 493 nm, emission 516 nm).

### 2.15. MitoSOX Assays

The production of mitochondrial superoxide was detected in live cells with MitoSOX Red dye (Thermo Scientific, Rockford, IL, USA). Cells were plated ($1\times 10^{4}$ cells/dish) onto 35-mm glass bottom dishes (MatTek, Ashland, MA, USA), transfected and incubated with 2.5 µM MitoSOX for 10 min at 37 °C. After washing 3 times in PBS, cells were incubated with a 1 μg/mL Hoechst staining solution (Thermo Scientific, Rockford, IL, USA) for 5 min at 37 °C. At least 3 images (20×) were taken per dish using a laser scanning confocal microscope (Leica TCS SP8-STED, Leica Microsystems, Mannheim, Germany) and Z-stacking. MitoSOX red fluorescence intensity was measured using Fiji/ImageJ software v1.54h.

### 2.16. Western Blotting

ARPE-19 cells were plated ($2\times 10^{6}$ cells/dish) onto 10-cm dishes and collected 48 h post-transfection. Proteins were extracted from cells by lysis in a lysis buffer solution containing M-PER, 1X protease inhibitor and 1X phosphatase inhibitor (Thermo Scientific, Rockford, IL, USA) for 1 h at 4 °C with inversion. Lysates were centrifuged at 13,000 rpm for 10 min at 4 °C. The aqueous protein solution was collected and concentrated using Amicon Ultra 10 k filters (Millipore, Sigma-Aldrich, Oakville, ON, Canada). Protein concentration was measured using Bradford assay (Sigma-Aldrich, Sigma-Aldrich, Oakville, ON, Canada) and 40 µg protein sample was loaded per well into 12% polyacrylamide gels, separated via electrophoresis and transferred at 4 °C overnight onto polyvinylidene difluoride (PVDF) membranes (Millipore, Bedford, MA, USA). Membranes were blocked in 5% BSA for 2 h and incubated with mouse anti-human p16^INK4a^ (1:500, Lifespan Biosciences, Shirley, MA, USA), mouse anti-human p21^Waf/Cip1^ (1:500, Lifespan Biosciences) or rabbit anti-human β-actin (1:5000, Novus Biologicals, Centennial, CO, USA) at 4 °C overnight. Next, membranes were incubated with HRP-conjugated anti-mouse or anti-rabbit (1:2500, Santa Cruz Biotechnology, Dallas, TX, USA) secondary antibodies for 1 h at room temperature. Protein bands were detected with an enhanced chemiluminescence (ECL) kit (Bio-Rad Laboratories, Hercules, CA, USA) using an ECL detection system (ImageQuant LAS 500, GE Healthcare Systems, Chicago, IL, USA). Blots were quantified via densitometry using ImageLab Software (V6.0.1 Bio-Rad Laboratories, Hercules, CA, USA) and normalized to β-actin.

### 2.17. Quantitative Real-Time PCR

Total miRNA was isolated from RPE/eyecups of mice or ARPE-19 cells using miRNAeasy kit (Qiagen, Toronto, ON, Canada). cDNA was prepared from total miRNA using the miRCURY LNA RT cDNA synthesis kit (Qiagen, Toronto, ON, Canada) as directed by the manufacturer. miRNA qPCR assays were performed in 96-well PCR plates (Bio-Rad Laboratories, Hercules, CA, USA). Total reaction volume of 10 µL per well contained 5 µL 2X miRCURY SYBR Green Master Mix, 3 µL 1 ng/µL cDNA (diluted 1:60), 1 µL specific primer mix (10µM) and 1 µL RNAse-free water. The reactions were performed on a LightCycler 96 Roche platform (Roche, IN, USA). Primers for let-7f and 103a-3p were obtained by Qiagen, Toronto, ON, Canada; primers are considered proprietary to the suppliers, not available to the public. miRNA expression was calculated by the 2^−ΔΔCt^ method and normalized to reference gene 103a-3p.

### 2.18. Statistical Analysis

Results are presented as means ± SD from at least three independent experiments performed with a minimum of 3 replicates per conditions. Two-tailed Student’s *t*-test was used to determine significant differences between two groups. One-way ANOVA followed by post-hoc Bonferroni test was used for the comparison of means from three or more groups. The threshold for statistical significance was set at *p* < 0.05.

## 3. Results

### 3.1. Let-7f Is Upregulated and Induces Oxidative Stress in RPE Cells

**Figure 1:**
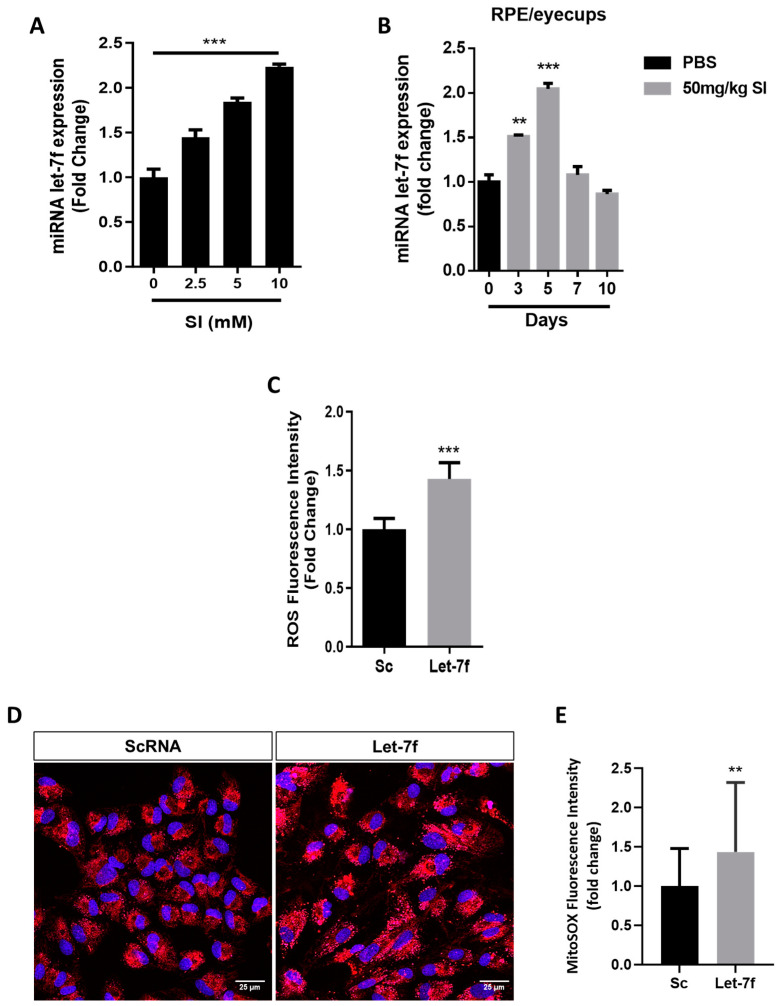
Oxidative stress is regulated by let-7f in RPE cells. (**A**) ARPE-19 cells were treated with increasing doses of SI (0, 2.5, 5 and 10 mM) for 48 h. The expression of let-7f miRNA was evaluated via RT-qPCR and expressed as a fold change versus untreated cells (0 mM). *** *p* < 0.001 vs. Untr. (**B**) Six eight-week-old female C57BL/6J mice were injected via the tail vein with 50 mg/kg SI or with vehicle (PBS) and sacrificed on days 0, 3, 5, 7 and 10. Total miRNA was isolated from RPE/eyecups and let-7f expression was quantified via RT-qPCR and expressed as fold change versus control retina (day 0, PBS). ** *p* < 0.01, *** *p* < 0.001 vs. CTL. (**C**) Let-7f overexpression was achieved by transfecting ARPE-19 cells with 50 nM let-7f mimic for 48 h and oxidative stress was then evaluated using CM-H_2_DCFDA assay to quantify the production of intracellular ROS. ROS fluorescence intensity was measured and presented as a fold change versus scramble control (Sc). *** *p* < 0.001 vs. Sc. (**D**) MitoSOX superoxide assay was used to detect mitochondrial ROS. Representative confocal images of live cells stained with MitoSOX (red) and Hoechst (nuclear DNA, blue). Scale bar = 25 µm. (**E**) Quantification of MitoSOX fluorescence intensity expressed as a fold change versus scramble control (Sc). ** *p* < 0.01 vs. Sc. All data are expressed as the mean ± SD (*n* = 3).

Let-7f expression has been shown to be upregulated in various age-related ocular disorders and to be modulated by oxidative stress [20,21,28,29]. We first investigated the expression of let-7f in RPE cells exposed to SI-induced oxidative stress. We demonstrated that let-7f expression increased dose-dependently in ARPE-19 cells treated with SI (Figure 1A). Additionally, we verified that the upregulation of let-7f was not limited to SI-induced oxidative stress: a similar dose-dependent increase in let-7f expression was observed in ARPE-19 cells treated with hydrogen peroxide (H_2_O_2_), another common inducer of oxidative stress (Figure S1A). To confirm our in vitro findings, we examined the expression of let-7f using the in vivo SI mouse model of oxidant-induced RPE damage. With H&E staining, we validated the model by demonstrating an overall thinning of the retinal layers along with the degeneration and formation of large drusen-like deposits at the RPE in mice injected with 50 mg/kg SI (Figure S2). Interestingly, the expression of let-7f in RPE/eyecups increased significantly at days 3 and 5 after SI injection, then returned to baseline levels at days 7 and 10 (Figure 1B). No significant change in let-7f expression was observed in the neural retina (Figure S1B), further suggesting that the relationship between let-7f and oxidative stress is localized to the RPE. Based on these results, we sought to determine whether the overexpression of let-7f could induce oxidative injury in healthy RPE cells. Oxidative stress was assessed at the intracellular and mitochondrial levels. ARPE-19 cells transfected with 50 nM let-7f miRNA showed approximately a 1.5-fold increase in the production of intracellular ROS than that observed in scramble (Sc) control transfected cells (Figure 1C). Similarly, the production of mtROS increased in let-7f overexpressing cells as revealed via MitoSOX red fluorescence intensity (Figure 1D,E). Overall, our findings suggest that let-7f plays a role in the regulation and induction of oxidative stress in RPE cells.

### 3.2. Let-7f Overexpression Promotes RPE Cellular Dysfunction

**Figure 2:**
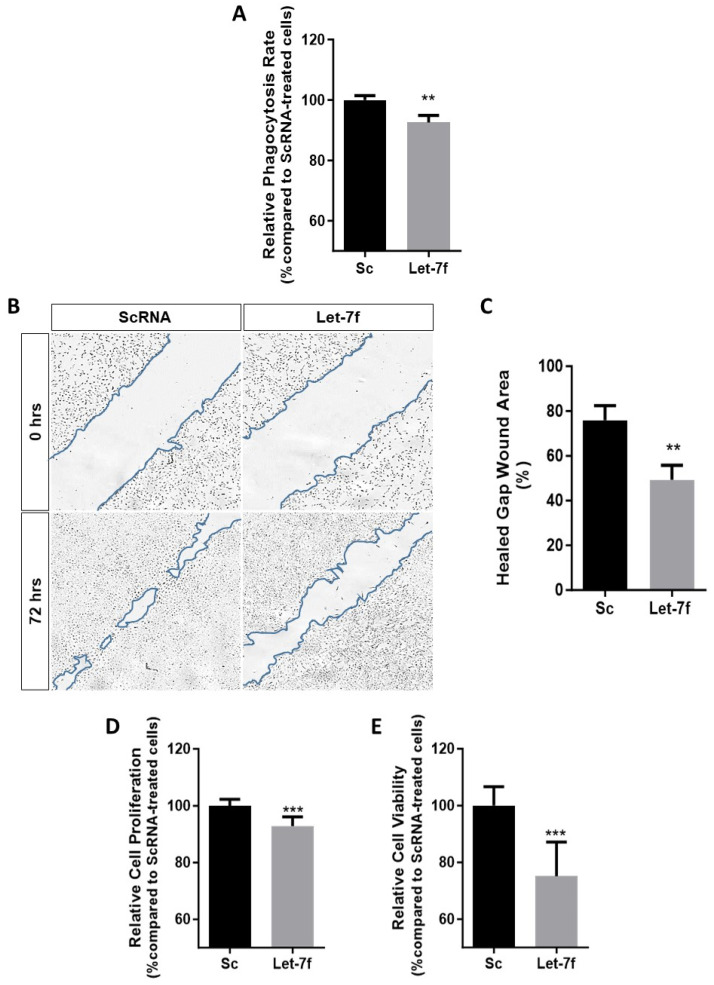
Let-7f induces cellular dysfunction in human retinal pigment epithelial cells. ARPE-19 cells were transfected with 50 nM let-7f mimic for 48 h and cellular functionality was examined. (**A**) Fluorescence intensity of engulfed POS-FITC was quantified over 6 h and expressed as a percentage compared to scramble control (Sc). (**B**) Representative images of scratch wound healing assay used to evaluate migration (4X). A scratch was made across confluent ARPE-19 cells and wound-healing was observed for 72 h. (**C**) Wound gap area was measured with ImageJ software v1.54h and the percent of healed gap wound area was calculated by the area reduction compared to time 0 and presented relative to scramble control (Sc). (**D**) Cellular proliferation was determined via [3H]-thymine incorporation assay and presented as percentage versus scramble control cells (Sc). (**E**) Cellular viability was analyzed using PrestoBlue^®^ assay and fluorescence intensity was measured and expressed as a percentage compared to scramble control cells (Sc). ** *p* < 0.01, *** *p* < 0.001 vs. Sc. All data are expressed as the mean ± SD (*n* = 3).

Having shown that the overexpression of let-7f induces oxidative stress in RPE cells (Figure 1), we hypothesized that its upregulation could also promote cellular dysfunction. A major role of the RPE is the efficient phagocytosis of shed photoreceptor outer segments (POS), a mechanism shown to be impaired by oxidative injury [30]. To explore the effects of let-7f on RPE phagocytic activity, we fed FITC-labeled POS (FITC-POS) to mature polarized ARPE-19 cells for a period of 6 h. The optimal POS incubation period was determined in preliminary testing (Figure S3). Although a 24 h incubation yielded a higher rate of phagocytosis, we opted for the 6 h incubation period due to evidence indicating that the inhibition of phagocytosis by oxidative stress is transient and can be restored after 24 h, giving rise to false negatives [31]. The fluorescence intensity of engulfed POS was measured and a reduction of approximately 10% in phagocytic activity was observed in ARPE-19 cells transfected with let-7f (Figure 2A). In addition, we examined the functional effects of let-7f on RPE cellular migration and proliferation—two important mechanisms that can be activated by the in vivo human RPE upon injury or cell death at the macula, as seen in geographic atrophy [32,33]. We observed that the overexpression of let-7f resulted in a significant decrease of both ARPE-19 cellular migration and proliferation, as revealed by scratch wound and ^3^H-thymidine incorporation assays, respectively (Figure 2B–D). Likewise, cellular viability was significantly reduced in cells overexpressing let-7f (Figure 2E). Comparably, preliminary tests using primary mouse RPE cells, which were characterized via RPE65 staining and successfully transfected with let-7f, revealed a dramatic decreased in cellular viability upon let-7f overexpression (Figure S4A–C). Our findings along with the detailed literature that has classified the let-7 family of miRNAs as major cell cycle regulators, shifted our interest towards investigating the possibility of let-7f-induced cell cycle arrest and senescence in RPE cells [15].

### 3.3. Overexpression of Let-7f Induces Senescence in RPE Cells

**Figure 3:**
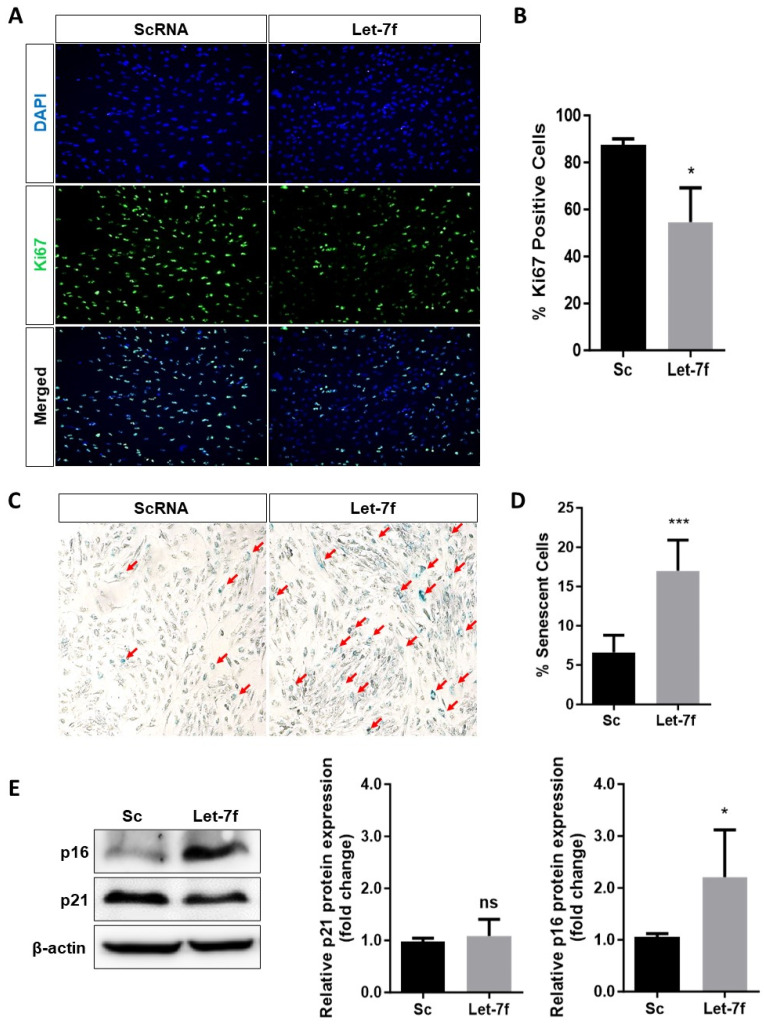
Let-7f induces a senescence phenotype in human retinal pigment epithelial cells. ARPE-19 cells were transfected with 50 nM let-7f mimic for 48 h and senescence was examined through various assays. (**A**) Proliferating cells were identified via immunofluorescent staining of Ki67 (green) positive cells and nuclei (DAPI, blue) (10×). (**B**) The percent of Ki67 positive cells was calculated based on the number of Ki67 positive cells divided by the total number of cells (DAPI positive) and expressed versus scramble control (Sc). (**C**) Representative images of senescence-associated β-galactosidase (SA-β-gal) staining used to detect senescent ARPE-19 cells. Senescent cells were identified by the development of blue coloration and an enlarged, flattened morphology as indicated by the red arrows (10×). (**D**) Percentage of senescent ARPE-19 cells was compared to scramble control (Sc). (**E**) The protein expression levels of cyclin-dependent kinase inhibitors p16^INK4a^ and p21^Waf/Cip1^ were detected via western blotting. Band densitometry was calculated using ImageLab Software and normalized to β-actin. Results were presented as a fold change compared to scramble control cells (Sc). * *p* < 0.05, *** *p* < 0.001 vs. Sc. ns: not significant. All data are expressed as the mean ± SD (*n* = 3).

Oxidative stress-induced senescence has been extensively associated with the development and progression of dry AMD [12]. To initiate our investigation, we examined the cellular expression of Ki67 nuclear protein marker, whose absence is indicative of cell cycle arrest at the G_0_ phase. Immunofluorescence staining revealed a substantial decrease in the number of Ki67 positive ARPE-19 cells when transfected with let-7f, suggesting an increase in cells arrested at G_0_ phase (Figure 3A,B). In addition to cell cycle arrest, a classic marker of cellular senescence is high activity of lysosomal β-galactosidase (β-gal) enzyme, referred to as senescence-associated β-gal (SA-β-gal), which can be detected and observed through SA-β-gal staining at pH 6 [27,34]. Consistently, overexpression of let-7f led to a dramatic increase in the number of senescent ARPE-19 cells, confirmed by the development of blue coloration upon SA-β-gal staining (Figure 3C,D). To further support our findings, we evaluated the protein expression levels of p16^INK4a^ and p21^Waf/Cip1^, two cyclin-dependent kinase inhibitors whose upregulations are established markers of senescence. Transfection of let-7f in ARPE-19 cells significantly increased the protein expression of p16^INK4a^ by approximately twofold, though no significant change in p21^Waf/Cip1^ protein expression was detected (Figure 3E). Interestingly, overexpression of let-7f in primary RPE cells led to the upregulation of p16^INK4a^ and p21^Waf/Cip1^ mRNA expression levels (Figure S4D). Collectively, our results strongly suggest that let-7f is involved in the induction of senescence in RPE cells.

### 3.4. Let-7f Inhibition Protects RPE Cells against SI-Induced Oxidative Injury

**Figure 4:**
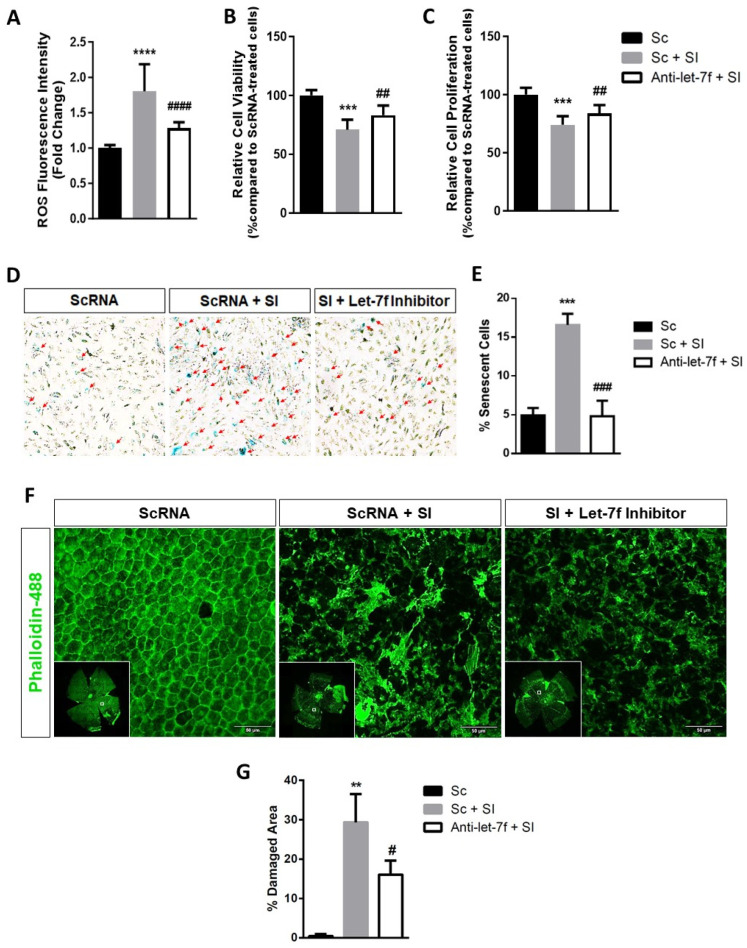
Inhibition of let-7f protects retinal pigment epithelial cells from sodium iodate-induced oxidative injury in vitro and in vivo. ARPE-19 cells were transfected with 20 nM let-7f antagomir (anti-let-7f) or with a scramble miRNA (Sc) and treated with 5 mM of SI for an additional 48 h. (**A**) The intracellular production of ROS was measured using CM-H_2_DCFDA assay and ROS fluorescence intensity was presented as a fold change versus scramble control (Sc). (**B**) Cellular viability was analyzed using PrestoBlue^®^ assay and fluorescence intensity was expressed as a percentage compared to scramble control cells (Sc). (**C**) Cellular proliferation was determined via [^3^H]-thymine incorporation assay and presented as a percentage versus scramble control cells (Sc). (**D**) Representative images of senescent cells detected via senescence-associated β-galactosidase (SA-β-gal) staining. Senescent cells were identified by the development of blue coloration and an enlarged and flattened shape morphology as indicated by the red arrows (10×). (**E**) Percentage of senescent ARPE-19 cells was compared to scramble control (Sc). (**F**) Six eight-week-old female C57BL/6J mice received an intravitreal injection of 1µg let-7f antagomir (anti-let-7f) or scramble miRNA (Sc) 24 h prior to tail-vein injection with 50 mg/kg SI. Mice were sacrificed a week later and RPE/eyecup wholemounts were prepared from dissected retina. Shown are fluorescent confocal images of RPE/eyecups stained with phalloidin-488 to visualize RPE monolayer morphology and damaged areas. Scale bar = 50 µm. (**G**) Percent damaged area was calculated using ImageJ software v1.54h and compared to scramble control (Sc) RPE/eyecups. ** *p* < 0.01, *** *p* < 0.001, **** *p* < 0.0001 vs. Sc. # *p* < 0.05, ## *p* < 0.01, ### *p* < 0.001, #### *p* < 0.0001 vs. Sc + SI. All data are expressed as the mean ± SD (*n* = 3).

We next investigated the protective potential of let-7f inhibition against the deleterious effects of oxidative injury on RPE cells. To this end, ARPE-19 cells were transfected with 20 nM let-7f antagomir (anti-let-7f), and subsequently treated with 5 mM SI. The concentration and duration of SI treatment were optimized to generate sub-lethal cytotoxic effects still capable of inducing oxidative injury, a model better representative of the chronic stress occurring in the in vivo RPE during the progression of dry AMD [35]. Previous studies have identified the EC50 of SI in ARPE-19 cells to be approximately 10 mM when exposed for 24 h and that an acute non-lethal dose at concentrations of 10 mM or lower allows the investigation of the early effects of oxidative injury in RPE cells [36,37]. The concentration of 5 mM (double-dose) was selected for reason that this dose displayed sub-lethal effects (Figure S5) yet was sufficient in inducing oxidative stress (Figure S6) and upregulating the expression of let-7f miRNA in ARPE-19 cells (Figure 1A). As expected, treatment with SI increased intracellular ROS production by almost twofold in ARPE-19 cells. Notably, let-7f inhibition significantly protected cells from this increase (Figure 4A). Additionally, the inhibition of let-7f in ARPE-19 cells helped to prevent the loss in viability and proliferation induced by pro-oxidant SI (Figure 4B,C). Furthermore, SA-β-gal staining revealed that let-7f inhibition impressively suppressed ARPE-19 cellular senescence brought on by SI (Figure 4D,E). To verify whether the protective potential of let-7f inhibition in vitro could be translated to the in vivo RPE, we used the well-established mouse model of SI-induced retinal degeneration which we described and validated (Figure S2). Mice received an intravitreal injection of a let-7f antagomir (anti-let7f) or a scrambled control mimic (Sc) 24 h prior to a systemic injection with 50 mg/kg SI. Seven days later, animals were sacrificed and RPE/eyecup wholemounts were prepared from dissected retina for immunofluorescent evaluation. Phalloidin staining showed no evident disruptions in the overall morphology and organization of the RPE monolayer in mice injected only with scrambled control. In contrast, treatment with SI completely disrupted the morphology of the RPE monolayer, causing degeneration and substantial areas of cell loss. Pre-treatment with let-7f antagomir (anti-let7f + SI) resulted in a significant reduction of damaged area though not a complete rescue of the RPE (Figure 4F,G). Together, our results have demonstrated the protective potential of let-7f inhibition against oxidative injury in RPE cells.

## 4. Discussion

Recently, miRNAs have become attractive therapeutic targets for AMD as they regulate multiple pathways in RPE cells and have been shown to play a role in the development and progression of this disorder [14,38,39,40]. Various studies have linked let-7f and its family to age-related ocular disorders as they are found to be highly expressed in aged lens, vitreous and retina and can also modulate oxidative stress and senescence in numerous cell types [20,21,22,28,29,41,42,43]. Despite the evidence, the functional effects of these miRNAs on RPE cells have remained largely uninvestigated. Thus, the present study examines the relationship between let-7f and oxidative stress in RPE cells, as well as its implication in cellular dysfunction.

Our initial in vitro results demonstrated that the expression of let-7f increased dose-dependently in ARPE-19 cells treated with the pro-oxidant sodium iodate (SI). We confirmed that this upregulation was a consequence of oxidative stress that was not limited to SI by demonstrating a similar response in cells treated with H_2_O_2_. These results are consistent with those reported by Li et al., demonstrating increased expression of let-7f in H_2_O_2_-treated human neuroblastoma cells associated with Alzheimer’s disease [44]. These findings are particularly noteworthy due to the many pathological features shared between AMD and Alzheimer’s disease [45,46].

Using an in vivo mouse model of SI-induced RPE degeneration, we observed an initial upregulation in let-7f expression, similar to the upregulation of miR-144 reported by Jadeja et al. using the same model [47]. Another such study demonstrated that following SI administration, rat plasma miR-124 expression levels increased significantly at days 3 and 4, and returned to baseline on day 8 [48]. Combined, the data suggests the existence of an initial miRNA response triggered by early oxidative injury in RPE cells, which could serve as useful targets in the search for predictive biomarkers and in the development of treatments for the early onset of AMD. Moreover, let-7f expression was found to be upregulated in photo-oxidative damaged rat retinas and in the plasma of monkeys exposed to cigarette smoke, two well-known risk factors associated with the development of AMD [22,49]. In line with these observations, we demonstrated that overexpression of let-7f in ARPE-19 cells promoted the production of intracellular and mitochondrial ROS. Oxidative stress has been reported to induce RPE cellular damage and dysfunction [6,7,9]. Indeed, we observed that cells transfected with let-7f exhibited decreased phagocytic activity, cellular migration, proliferation and viability. Interestingly, a recent study by Yang et al. has demonstrated that SI induces oxidative stress and promotes wound healing of ARPE-19 cells [50]. On the other hand, our results show that overexpression of let-7f induces oxidative stress but instead inhibits wound healing of ARPE-19 cells. After careful evaluation of both studies, many factors can be identified as contributors to this discrepancy. A major difference is the exposure of SI to ARPE-19 cells. Yang et al.’s team reports increased cellular migration after treatment with 2 mM SI for 24 h. According to our findings, treatment with 2 mM SI did not lead to the significant overexpression of let-7f that was achieved using a dose of 5 mM, and may be the reason for the contrasting results. Additionally, Yang et al. examined the protein expression of several EMT markers as well as signaling factors of the antioxidant response element, and reported increased expression upon SI treatment. However, the majority of their findings also revealed that maximum protein expression was achieved at 1.5 mM SI and decreased at 2 mM, which suggest the possibility that at higher doses, such as the 5 mM used in our study, migration may be inhibited. Overall, our findings provide strong evidence for a relationship between let-7f and oxidative stress in the RPE.

Cellular senescence, both stress- and age-induced, has emerged as a key player in the development and progression of age-associated disorders, such as AMD [12,51]. Numerous studies have shown that oxidative stress at the RPE can induce cellular senescence, further exacerbating the effects of the injury [11,52,53]. Through the increase in Ki67-negative and SA-β-gal-stained cells, along with the upregulation of senescent protein marker p16^INK4a^, our data demonstrates that overexpression of let-7f induces RPE cellular senescence. Surprisingly, the expression level of senescent protein marker p21^Waf/Cip1^ remained essentially unchanged. Such an observation could be attributed to the fact that activation of p21^Waf/Cip1^ in senescent cells is transient, whilst p16^INK4a^ remains constitutively expressed to maintain the senescent state [54]. Our findings are consistent with those of previous works demonstrating that let-7f and other members of its family can induce cell cycle arrest at the G_0_/G_1_ phase [16,55,56]. Specifically, let-7f is found to be upregulated in both oxidative stress and age-induced senescent cells [18,42,57,58]. Together, our findings strongly suggest that let-7f may play an important role in both age-associated and stress-induced senescence of the RPE.

We next examined the protective potential of let-7f inhibition against SI-induced oxidative injury in RPE cells. Our in vitro findings demonstrated that the inhibition of let-7f proved capable of protecting ARPE-19 cells against ROS overproduction, cellular dysfunction and the induction of senescence generated by exposure to SI. Furthermore, our in vivo observations are consistent with that of the literature in revealing RPE monolayer degeneration and severe morphological disruption following systemic injection of SI [24,25,59,60]. Pre-treatment with a let7f inhibitor significantly decreased the areas of damaged or lost RPE cells but failed to preserve the overall structure of the RPE monolayer. The resultant incomplete protection could be due in part to other members of the let-7 family compensating for the suppression of let-7f. More importantly, miRNA networks are highly complex and involve multiple players that can act simultaneously on various cellular pathways, emphasizing both the difficulty and the importance of understanding the role of every piece of the miRNA puzzle.

Our study is the first to report a protective effect against oxidative injury in RPE cells through the inhibition of let-7f. Interestingly, this finding is contradictory to that of another in which let-7f protected against H_2_O_2_-induced oxidative damage in neuroblastoma cells, despite an initial upregulation in let-7f expression following oxidative injury as was observed in our study [44]. This discrepancy may be attributed to differences in cell type and chemical stressor as well as their model’s focus on inducing apoptotic damage by oxidative stress rather than the sub-lethal stress we chose to examine, likely activating different response pathways in RPE cells. Hence, a deeper understanding of the functional and regulatory roles played by this miRNA in the RPE is crucial.

## 5. Conclusions

Our findings successfully demonstrate the implication of let-7f in the degeneration and dysfunction of RPE cells through the regulation of oxidative stress and senescence. Further mechanistic studies and investigation towards this relationship could provide novel insights into the pathogenesis, diagnosis and future treatments of dry AMD.

## Data Availability

The data are contained within this article.

## Supplementary Materials

The following supporting information can be downloaded at: https://www.mdpi.com/article/10.3390/antiox13060646/s1, Figure S1. Oxidative stress upregulates let-7f expression in RPE cells. Figure S2. Hematoxylin & eosin (H&E) stained retinal sections demonstrate time-dependent retinal injury in the sodium iodate-induced mouse model of dry AMD. Figure S3. ARPE-19 time-dependent phagocytosis of photoreceptor outer segments (POS). Figure S4. Overexpression of let-7f induces cellular dysfunction in primary RPE cells. Figure S5. Sodium iodate (SI) decreases RPE cellular viability in a time and dose-dependent manner. Figure S6. Sodium iodate (SI) increases ROS production in RPE cells in a time and dose-dependent manner. Table S1: Sequences of quantitative RT-PCR primers.
