# Supplementary material for: The microRNA Let-7f Induces Senescence and Exacerbates Oxidative Stress in Retinal Pigment Epithelial Cells
Source: Antioxidants (Basel). 2024 May 25;13(6):646. doi: 10.3390/antiox13060646 (PMC11200580; doi:10.3390/antiox13060646)
Supplement: Supplementary file 1 [file antioxidants-13-00646-s001.zip › antioxidants-3009945-supplementary.pdf]

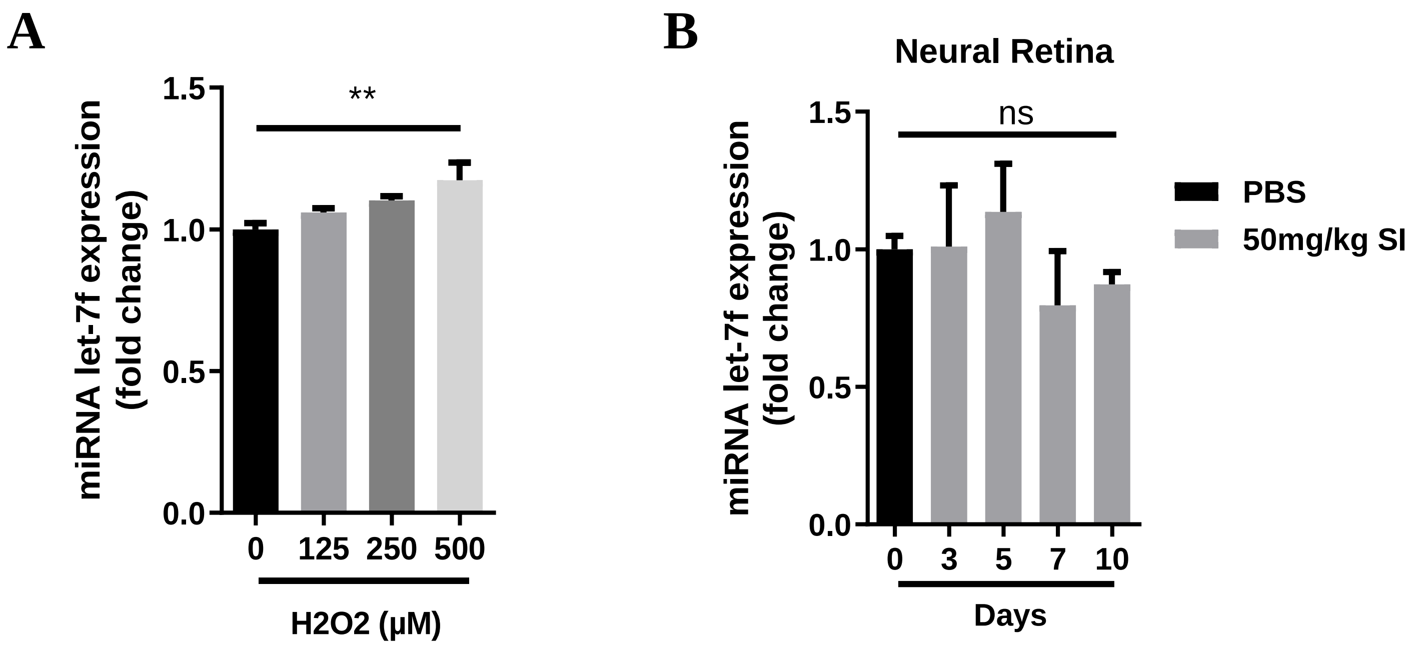

Figure S1. Oxidative stress upregulates let-7f expression in RPE cells. **(A)** Human retinal pigment epithelial (ARPE-19) cells were treated with increasing doses of H_2O_2 (0, 125, 250 and 500 µM) for 24 h. Total miRNA was isolated from cells and expression of let-7f was evaluated by RT-qPCR. Expression levels are presented as a fold-change versus untreated cells (0 µM). $^{**}p < 0.01$ vs Untr. **(B)** Six-eight-week-old female C57BL/6J mice received a 50 mg/kg SI tail-vein injection and were sacrificed on days 0, 3, 5, 7 and 10 to isolate total miRNA from the neural retina. Let-7f expression was evaluated by RT-qPCR and expressed as a fold-change versus control mice (day 0, PBS). ns: not significant.

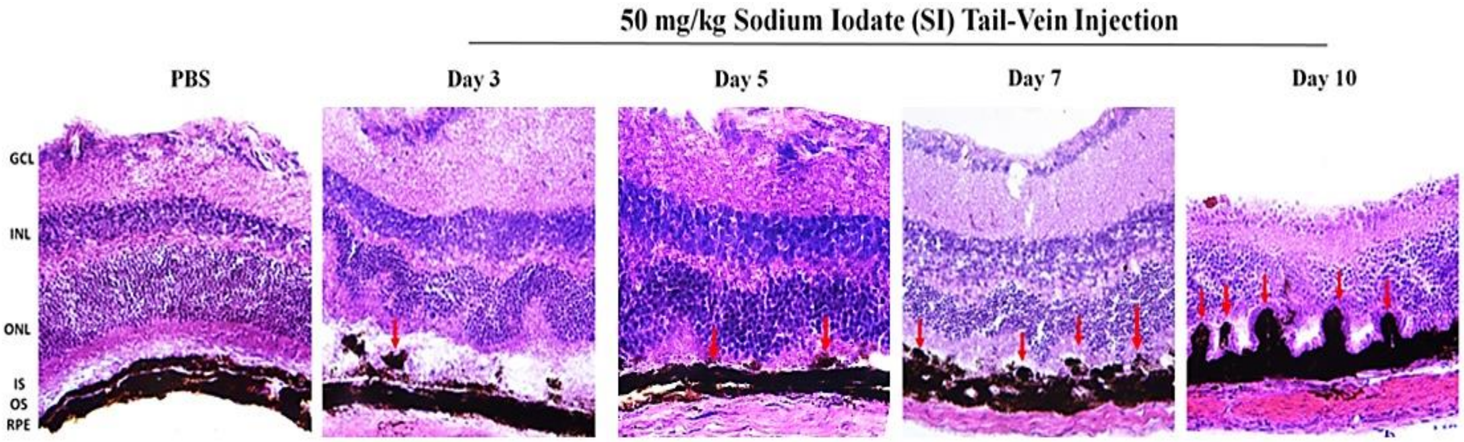

Figure S2. Hematoxylin & eosin (H&E) stained retinal sections demonstrate time-dependent retinal injury in the sodium iodate-induced mouse model of dry AMD. Six-eight-week-old female C57BL/6J mice were injected by tail-vein with 50 mg/kg SI or with vehicle (PBS) and sacrificed on days 0, 3, 5, 7 and 10. Eyes were collected and retinal cryosections (12 µm) were prepared and stained with H&E. Areas with apparent lipofuscin accumulation and drusen formation are indicated by red arrows. Sections were imaged at 20X using a bright-field microscope. GCL: ganglion cell layer; ONL: outer nuclear layer; INL: inner nuclear layer; IS: inner segment; OS: outer segment; RPE: retinal pigment epithelium.

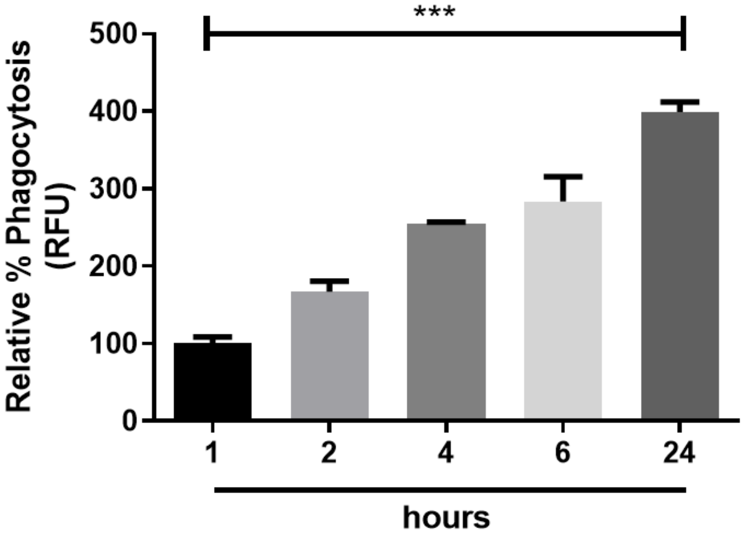

Figure S3. ARPE-19 time-dependent phagocytosis of photoreceptor outer segments (POS). Mature ARPE-19 cells were incubated with FITC-labelled POS for the indicated time points to determine optimal incubation period. Fluorescence intensity of engulfed POS was measured and expressed as a percentage compared to 1h-treated cells. ***$p < 0.001$ vs. t=1h.

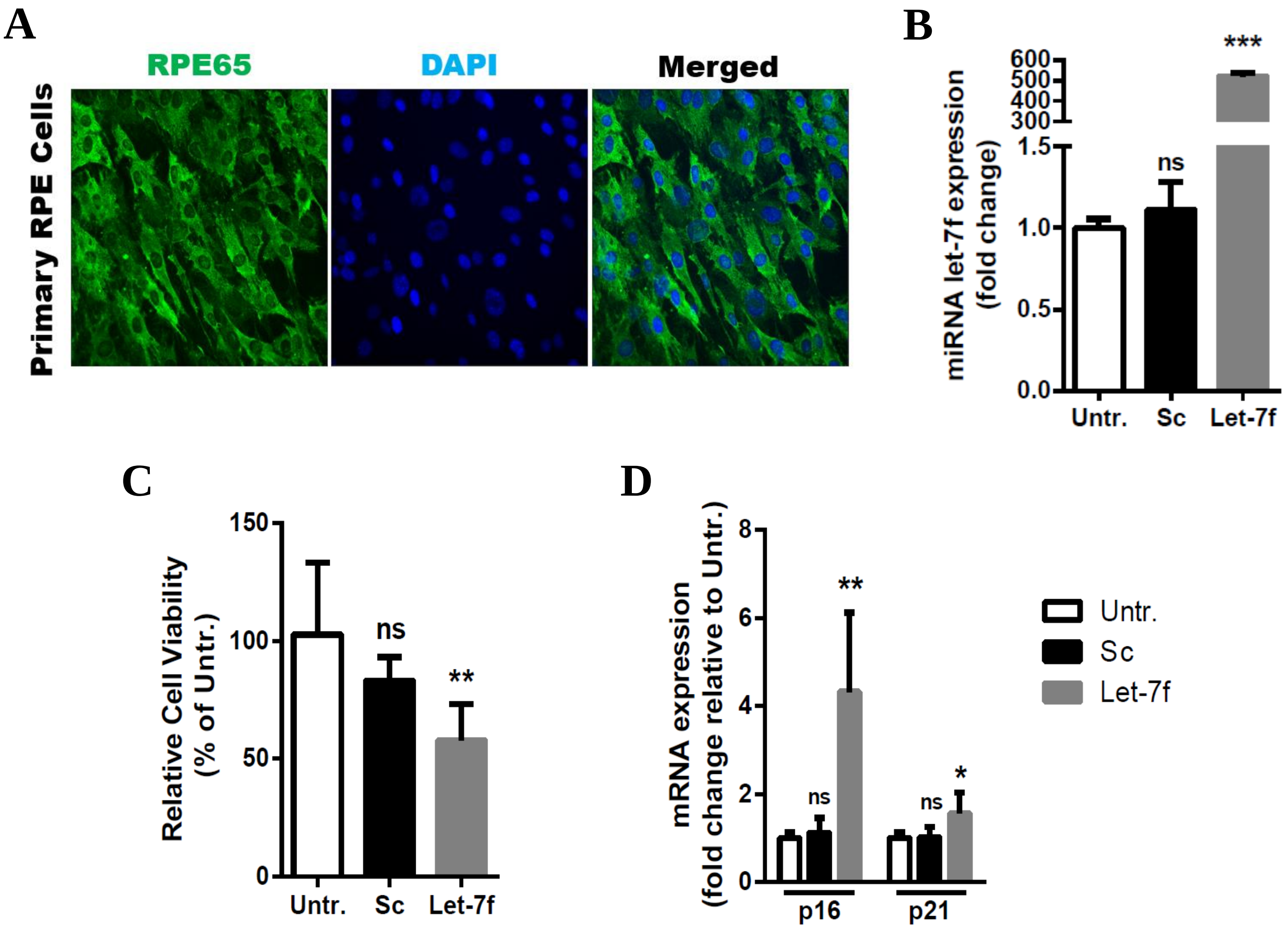

Figure S4. Overexpression of let-7f induces cellular dysfunction in primary RPE cells. **(A)** Primary RPE cell cultures were isolated from C57BL/6J mouse pups and their identity was validated by immunofluorescent staining of RPE-specific marker RPE65 (green) and DAPI (blue) (10X). **(B)** Upregulation of let-7f miRNA in primary RPE cells was confirmed by evaluating let-7f expression using RT-qPCR in untreated (Untr.) cells and in cells transfected with scramble miRNA (Sc) or 50 nM of a let-7f mimic. Results are expressed as a fold-change versus untreated cells. ***$p < 0.001$ vs. Untr. **(C)** Cellular viability was analyzed using PrestoBlue® assay. Fluorescence intensity was measured and presented as a percentage compared to untreated (Untr.) cells. **(D)** mRNA expression levels of cyclin-dependent kinase inhibitors $p16^{INK4a}$ and $p21^{Waf/Cip1}$ in primary RPE cells were detected by RT-qPCR. Expression levels are presented as a fold-change versus untreated (Untr.) cells. *$p < 0.05$, **$p < 0.01$, ***$p < 0.001$ vs. CTL. ns: not significant.

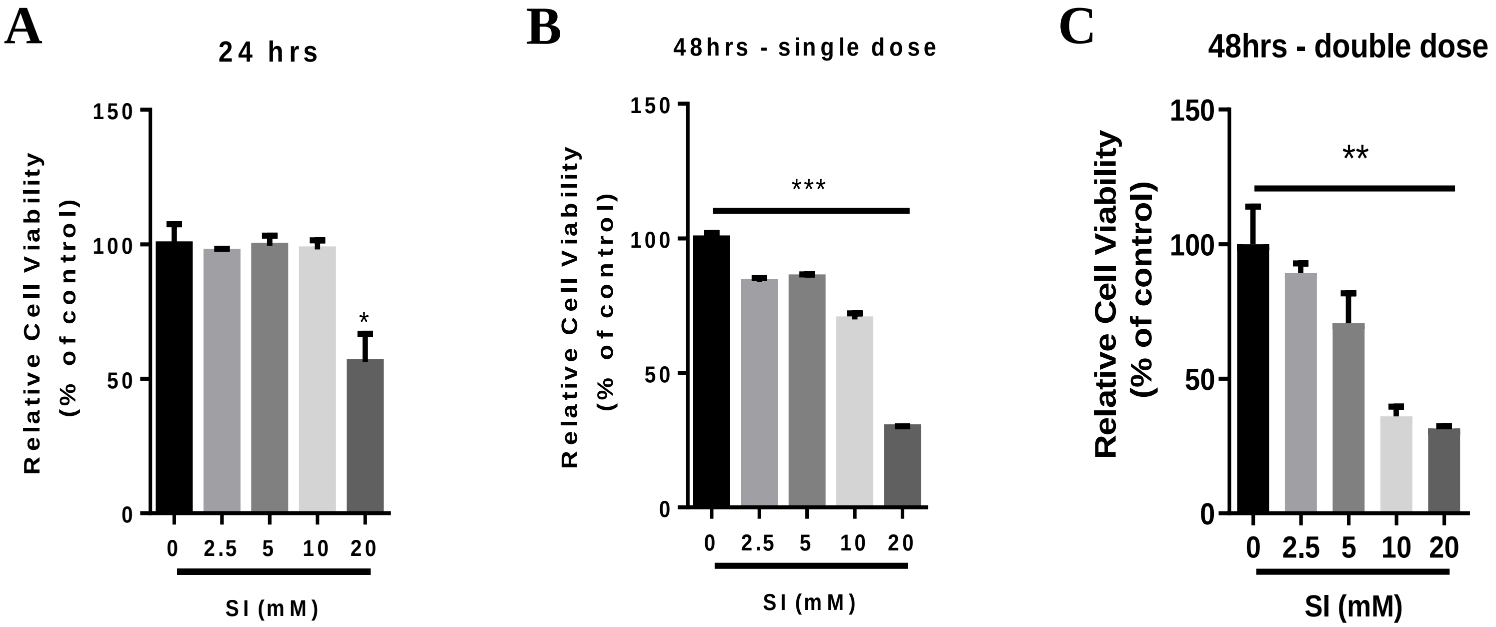

Figure S5. Sodium iodate (SI) decreases RPE cellular viability in a time and dose-dependent manner. ARPE-19 cells were treated with increasing doses of SI (0, 2.5, 5, 10 and 20 mM) for either **(A)** 24 h or 48 h with a **(B)** single or **(C)** double dose. Cellular viability was analyzed using PrestoBlue® assay and fluorescence intensity was measured and presented as a percentage compared to control cells (0 mM). *$p < 0.05$, **$p < 0.01$, ***$p < 0.001$ vs. CTL.

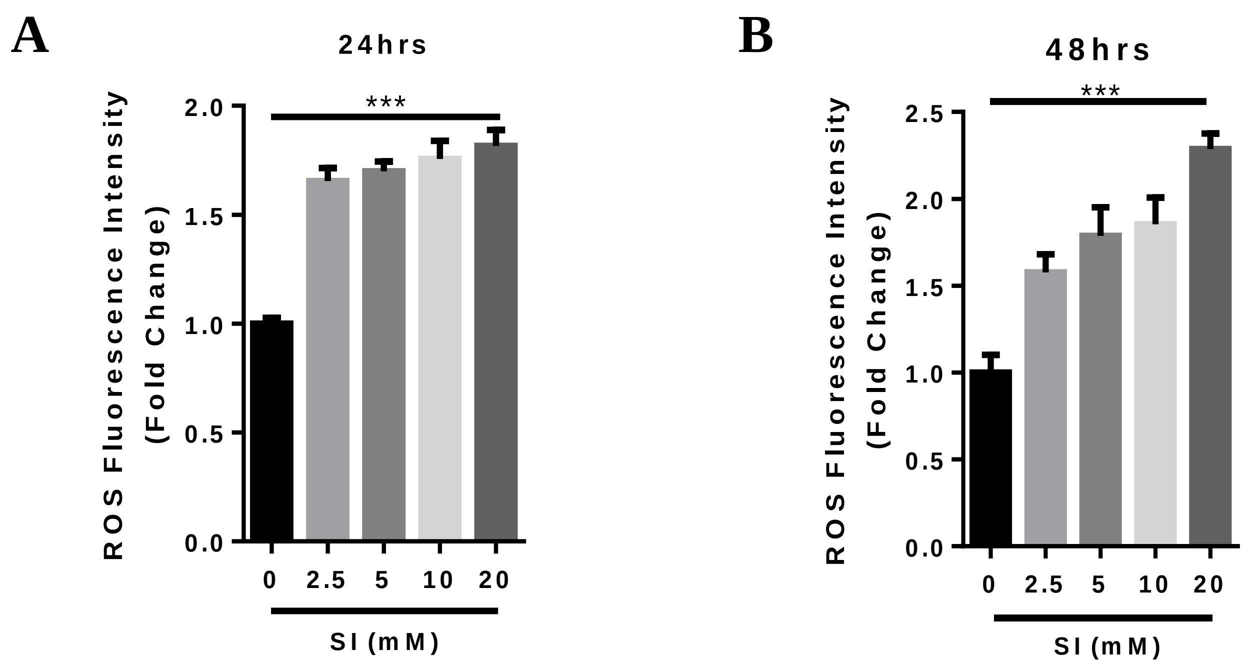

Figure S6. Sodium iodate (SI) increases ROS production in RPE cells in a time and dose-dependent manner. ARPE-19 cells were treated with increasing doses of SI (0, 2.5, 5, 10 and 20 mM) for **(A)** 24h or **(B)** 48h (double dose). Intracellular production of ROS was evaluated using CM-H_2DCFDA assay. ROS fluorescence intensity was measured and presented as a fold-change versus to control cells (0 mM). ***$p < 0.001$ vs. CTL.
